# Sexual Enhancing Effect of* Anacardium occidentale* in Stress-Exposed Rats by Improving Dopaminergic and Testicular Functions

**DOI:** 10.1155/2018/6452965

**Published:** 2018-10-25

**Authors:** Jintanaporn Wattanathorn, Thawatchai Prabsattroo, Pichet Somsapt, Opass Sritragool, Wipawee Thukham-mee, Supaporn Muchimapura

**Affiliations:** ^1^Department of Physiology, Faculty of Medicine, Khon Kaen University, 40002, Thailand; ^2^Integrative Complementary and Alternative Medicine Research and Development Center, Khon Kaen University, 40002, Thailand; ^3^Division of Nuclear Medicine, Department of Radiology, Faculty of Medicine, Khon Kaen University, Khon Kaen, Thailand

## Abstract

In this study, we aimed to assess the effect and possible underlying mechanism of* Anacardium occidentale* leaves extract on male sexual behaviors in stress-exposed rats. Male Wistar rats were orally given* A. occidentale* extract at doses of 25, 100, and 200 mg/kg BW before 12-hour-immobilization exposure for 14 days. Sexual behaviors, serum testosterone and corticosterone levels, TH-positive cells density in nucleus accumbens (NAc) and ventral tegmental area (VTA), MAO-B activity in NAc and medial preoptic area (MPOA), testis histology together with phosphodiesterase type-5 ( PDE-5) activity, and endothelial nitric oxide synthase (eNOS) expression in penis were evaluated after treatment. All doses of extract improved male sexual behaviors, suppressed MAO-B in NAc, enhanced TH-positive cells density in NAc, suppressed PDE-5 in penis, and enhanced interstitial cell of Leydig. The increase of serum testosterone, TH-positive cells density in VTA, eNOS expression in penis, and the decreased serum corticosterone were observed at some doses. Therefore, the sexual enhancing effect of extract occurred mainly via the improved dopaminergic and testicular functions. PDE-5 suppression in penis also played the role especially in the increased intromission behavior. Therefore,* A. occidentale* leaves extract is the potential protective agent against sexual dysfunction. However, further researches are necessary.

## 1. Introduction

Stress is regarded as one of the important factors of sexual dysfunction and infertility. It has been reported that stress can induce male sexual disturbances expressed as erectile dysfunction, ejaculatory disorders, loss of libido and a decrease in the frequency of intercourse [1], and a reduction of spermatogenesis [2]. The effects occur partly via the suppression of the hypothalamic-pituitary-gonadal (HPG-axis) axis [2]. In addition, a recent study also demonstrated that the dopaminergic system also contributed an important role in the regulation of male sexual function [3]. Therefore, the male sexual dysfunction appears to be a complex, multifactorial condition.

Repeated exposure to immobilization/restraint stress has been recognized as the one of most commonly used stress inducer models in rodents and can produce the deleterious effects on numerous systems including sexual dysfunction as those observed in humans [4]. It has been reported that repeated exposure to immobilization stress induces the suppression of testosterone secretion [5], sexual motivation, testicular maturation, and spermatogenesis [6, 7]. However, our pilot study has shown that exposure to immobilization stress failed to produce the significant changes of aforementioned parameters. Only some parameters such as intromission can be observed. The repeated exposure to 12-hour-immobilization stress can produce the significant changes of male sexual behaviors, serum testosterone, and spermatogenesis and these findings are in agreement with the previous study [8–10]. Since this model successfully produces the impairment of all significant male sexual parameters, the repeated exposure to 12-hour-immobilization stress has been used as the model to induce sexual dysfunction in this study. However, the observed changes can possibly occur not only via a direct effect of immobilization stress but also via the stress induced by dehydration and/or hunger.

The current medication therapeutic strategies still focus only on the improvement of HPG-axis. In addition, most of them are expensive and produce serious side effects [9–11]. Due to the limitation of efficacy and high expenditure cost of the current therapeutic agent, the novel protective agent against sexual dysfunction induced by stress which is effective and easy to achieve is required. Medicinal plants and plant-derived substances have been long term used for treating sexual dysfunction. They can improve libido, sexual potency, and sexual pleasure [12–14].* Anacardium occidentale* or Mamuang Himapan in Thai or Cashew, a native plant in Brazil, is widely cultivated in the southern, southeastern, and the eastern parts of Thailand. Leaf of this plant is consumed as food in Thailand. However, it has been reported that* A. occidentale* leaf is also used for treating genital problems, venereal disease, and sexual impotence in Brazil and Peru [15–17]. Although* A. occidentale* is reputed for sexual enhancing effect, no scientific evidence concerning this point is available. Therefore, this study was carried out to determine the effect of* A. occidentale* leaves extract on male sexual behaviors of stress-exposed rats. The possible underlying mechanisms were also further explored.

## 2. Materials and Methods

### 2.1. Plant Collection and Extraction

The fresh leaves of* Anacardium occidentale *(*A. occidentale*) Lin leaves (family; Anacaceae) were collected from Phuket province Thailand. The plant specimen was authenticated by Associate Professor Dr. Panee Sirisa-ard, Faculty of Pharmacy, Chiangmai University, Thailand. The voucher specimen was deposited at Integrative Complementary Alternative Medicine Research and Development Center, Khon Kaen University. Alcoholic extract (95%) of* A. occidentale* leaves was prepared by maceration technique. In brief, the leaves were dried in an oven at 45°C and powdered in a knife and hammer mill. The dried powder was then extracted with 95% ethanol at a ratio of 1:2 (w/v). The extract was filtered by filter paper Whatman number 1 and evaporated to dryness under reduced pressure by using rotary evaporator. The percentage yield of the extract was 17.32%. The concentration of total phenolic compounds was 102.963±0.006 mg gallic acid equivalent (GAE)/g extract. In addition, the contents of gallic acid and quercetin were 7.771±0.003 mg GAE/mg extract and 0.617±0.0001 mg QE/mg extract, respectively.

### 2.2. Experimental Animals

Healthy, sexually mature, male Wistar rats weighing 250-350 g and female rats weighing 200-250 g were obtained from National Laboratory Animal Center, Salaya, Nakhon Pathom province, Thailand. They were housed in group of 6 per cage in standard metal cages at 24±2°C on 12:12 h light-dark cycle. All animals were given access to food and water* ad libitum*. The experiments were performed to minimize animal suffering in accordance with the internationally accepted principles for laboratory use and care of European Community (EEC directive of 1986; 86/609/EEC).The experimental protocols were approved by the Institutional Animal Care and Use Committee.

### 2.3. Experimental Protocol

Total 42 male rats (n=6/group) were randomly divided into various groups as described below.

  Group I: Naïve control (nonstress group): rats were not given any substance.
  Group II: Vehicle plus stress: vehicle (distilled water) was administered to all rats at 45 minutes before the exposure to 12-hour-restraint stress exposure.
  Group III: Sildenafil: this group was served as positive control based on the sexual enhancing effect of Sildenafil citrate. Sildenafil citrate at dose of 5 mg/ kg was administered to the stress exposed rats at 45 minutes prior to the copulatory study.
  Group IV: Tianeptine: animals in this group were also used as positive control based on its antistress effect. All stress exposed rats were given Tianeptine at dose of 15 mg/ kg at 45 minutes prior to the copulatory study.
  Groups V-VII:* A. occidentale* leaves extract treated groups: the hydroalcoholic extract of* A. occidentale* leaves extract at doses of 25, 100, and 200 mg/kg was administered to all rats at 45 minutes before subjecting to restraint stress.

In this study, all assigned substances were orally given and stress exposure was performed by restraining rat for 12 hours (Based on the pilot data which demonstrated that this duration induced sexual dysfunction together with the decreased testosterone level and spermatogenesis). The treatments and the stress exposure were carried out once daily at a period of 14 days. Following stress exposure, the animals were subjected to 3-hour-refreshment period before subjecting to the sexual behavior evaluation. The sexual behaviors assessments were performed by an experienced observer blind to treatment at room temperature between 9.00 p.m. to 12.00 a.m. after the single intervention and 7 and 14 days of treatment.

In order to assess the sexual behaviors, estrous female rats were paired with male treated with the assigned substances. Female rats were induced to estrous by sequential administration of estradiol benzoate (Sigma, St. Louis, MO) at dose of 2 *μ*g kg/BW and progesterone (Sigma, St. Louis, MO) at dose of 500 *μ*g kg/ BW via subcutaneous route at 48 hours and 6 hours prior to the determination of copulatory behavior [18]. Sexual behaviors were monitored in a separate room for 3 hours in a clear plastic box for 30 minutes at the start of first hour whereas the whole duration of observation was recorded by digital video recording [19]. The assessed sexual parameters were described below:

Mounting frequency: the frequency of mounts without intromission from the time of introduction of the female until ejaculation.

Intromission frequency: the frequency of intromissions from the time of introduction of the female until ejaculation.

Mount latency: the time interval between the introductions of the female to the first mount by the male.

Intromission latency: the interval from the time of introduction of the female to the first intromission by the male.

Ejaculation frequency: the frequency of ejaculation which characterized by longer, deeper pelvic thrusting, and slow dismount followed by a period of inactivity.

Ejaculation latency: the time interval between the first intromission and ejaculation [20].

### 2.4. Stress Induction

A 12-hour-stress exposure was carried out between 6.00 a.m. to 6.00 p.m. for 14 days. In brief, all animals were placed in a transparent perforated plastic tube, 20 cm long, and 7 cm in diameter and the tubes were closed with Plexiglas lids. The animals fit tightly into the restrainers so it was not possible for them to turn around. The induction of stress was performed at the same period every day throughout the study period. Naïve control rats were maintained at room temperature with free access to food and water and were not subjected to any procedure until the experiment.

### 2.5. Determination of Phosphodiesterase Type 5 (PDE-5)

The determination of PDE-5 was carried out by using PDE-Glo™Phosphodiesterase Assay kit (Promega). The penis was isolated and prepared as homogenate by using lysate RIPA buffer. Then, the homogenate was subjected to a 14,000*g *–centrifugation at 4°C for 15 minutes. The supernatant was separated and served for the determination of PDE-5 activity. The PDE-Glo™ phosphodiesterase assay was carried out in a 96-well plate. The assay was performed according to the guidelines of the kit. In brief, the penis was incubated with cyclic guanosine monophosphate (cGMP) substrate in reaction buffer until the phosphodiesterase reaction was complete. PDE-Glo™ termination buffer was incubated with PDE detection solution containing adenosine triphosphate (ATP) and protein kinase A (PKA). The amount of ATP consumed by this reaction which is directly correlated with the cGMP level was evaluated using the luciferase-based Kinase-Glo reagent. Following a 10-minute incubation period at room temperature, the optical density of the sample was determined using a SpectraMax® L microplate luminometer (MDS AT (US) Inc.) and expressed as relative light units (RLUs) and as a percentage of the control [20].

### 2.6. Determination of Monoamine Oxidase Type B

At the end of experiment, all rats were killed to determine monoamine oxidase type B activity in medial preoptic area (MPOA) and nucleus accumbens (NAc) by using the peroxidase-linked photometric assay [21]. In brief, 2.75 mL Tris-buffer (0.1 M, pH 7.4) and 100 *μ*L of 0.1 M benzylamine were mixed in quartz cuvette which was placed in double beam spectrophotometer and followed by the addition of 150 *μ*L solution of brain homogenate to initiate the enzymatic reaction. The change in absorbance was recorded at wavelength of 249.5 nm for 5 min against the blank containing Tris-buffer and 5-hydroxytryptamine. MAO-B activity was calculated after 30 minute incubation [20].

### 2.7. Determination of Testosterone and Corticosterone Levels

The venous blood of each animal was collected from tail vein and kept on ice. Then, it was subjected to a 2000 g-centrifugation at 4°C for 15 minutes. The obtained serum was kept at −80°C until used. Testosterone levels were measured using a radioimmunoassay (RIA) kit (TESTO-CT2, Cisbio International, France) whereas corticosterone levels were measured using Corticosterone Double Antibody Radioimmunoassay Kit (MP Biomedicals) for the quantitative determination of corticosterone in rat and mice serum. The amount of labeled testosterone bound to the antibody was inversely related to the amount of unlabeled testosterone present in the sample. The remaining radioactivity bound to the tube was measured with Gamma scintillation counter which calibrated for Iodine-125. The results were showed as ng/ml.

### 2.8. Tyrosine Hydroxylase Immunohistochemistry Study

The brains were removed and fixed with 4% paraformaldehyde overnight and then postfixed with cryoprotective solution (30% sucrose in 0.1 M PBS) until they were sunk. Brain coronal sections containing nucleus accumbens and ventral tegmental area (VTA) at 10 *μ*m thick were prepared by using a cryostat sectioning (Micro HM 525). The density of tyrosine hydroxylase positive cells was determined with immunohistochemistry. In brief, the sections were retrieved antigen by heat-induced epitope retrieval (HIER) method in Tris-EDTA retrieval buffer (containing 0.05% tween 20, pH 9.0) by using microwave for 20 minutes. Then, the retrieved sections were incubated with 3% H_2_O_2_ in PBS (containing 0.1% sodium azide) at room temperature for 60 minutes in order to block endogenous peroxidase and incubated with 1% normal goat serum in PBS (containing 0.05% BSA, 0.1% gelation, and 0.3% Triton X-100) at room temperature for 30-60 minutes to block nonspecific binding. Following this process, the sections were incubated with primary monoclonal mouse anti TH (1:400; Sigma) in PBS-T (containing 1% normal goat serum and 0.3% Triton X-100) overnight at 4°C. Sections were washed twice for 5 minutes each with cool PBS and then incubated with Dako REAL™ EnVision™/HRP, Rabbit/Mouse (ENV) at room temperature for 2 hours. Then, they were incubated with Dako REAL™ DAB+Chromogen in Dako REAL™ Substrate Buffer at room temperature for 10 minutes. Sections were dehydrated with serial dilution of alcohol from 70 to 100%. Finally, they were cleaned with xylene, dried, and coverslipped using MERCK KGaA mounting medium. Images were captured in the area of nucleus accumbens (NAc; Bregma +1.6 mm, Interaural 10.6 mm) and medial preoptic area (mPOA; Bregma -0.5 mm Interaural 0.5 mm) according to the stereotactic rat brain atlas of Paxinos and Watson by using the Olympus Cover-018 light microscope. TH-immunopositive (IR) neurons were counted in mPOA and NAc by using the ImageJ software (Version 1.48V, National Institutes of Health). In brief, the area containing a brain section was defined, focus was adjusted manually, the area was scanned, and individual images in the area were stitched together automatically. The density values were corrected with nonspecific background from the cortex. The data are expressed as a pixel density.

### 2.9. Determination of Endothelial Nitric Oxide Synthase

The expression of endothelial nitric oxide synthase (eNOS) in penis was assessed via western blot assessment. In brief, homogenate of penis was prepared by using RIPA buffer with protease inhibitors. Then, the homogenate was subjected to a 14,000 g-centrifugation at 4°C for 20 minutes. The supernatant was collected and measured the level of protein by using NANO drop Spectrophotometers. Equal amounts of protein (100 *µ*g) were separated by 8% SDS-polyacrylamide gel electrophoresis and protein bands were transferred to a polyvinylidene difluoride (PVDF) membrane (Bio-Rad Laboratories, Hercules, CA). Following this process, the membranes were subjected to a 5 minute-rinsing in 0.05% Tris-buffer saline with Tween-20for 3 times. Then, the membranes were blocked with 5% skim milk in Tris-buffer saline with 0.05% Tween-20) and incubated overnight in primary antisera against eNOS (1:500). The membranes were then incubated with horseradish peroxidase-linked secondary antibody (1:4,000) for 1 hour at room temperature. Then the signal was enhanced with a Thermo Scientific™ Pierce™ ECL Substrate chemiluminescence kit (Pierce™ ECL Western Blotting). Images were acquired by ImageQuant LAS 4000, GE Healthcare. Band densities were quantified with NIH-ImageJ (Version 1.48V; National Institutes of Health, USA). The PVDF was reprobed with the beta actin antibody (1:2,000) as a loading control.

### 2.10. Testicular Histological Study

The testes were removed, fixed with 4% paraformaldehyde for 24 hr, and postfixed with 30% sucrose. They were prepared as a 10 *μ*m thick sections and stained with hematoxylin and eosin (H&E). In brief, the sections were washed with PBS for 5-10 minutes and stained with Weigert's hematoxylin for 10-15 minutes. Slides were washed twice with PBS for 1 minute and then stained with Eosin Y for 45 seconds. The stained slides were washed and dehydrated with serial dilution of ethanol and cleaned with xylene. The sections were dried and coverslipped using MERCK KGaA mounting medium. Histomorphology was determined using light microscope..

### 2.11. Statistical Analysis

All data were expressed as mean ± SEM value. The significant difference among various groups was evaluated by ANOVA and followed by LSD test by using the SPSS software package for Windows. The statistical difference was regarded at p-value < 0.05.

## 3. Results

### 3.1. Effect of* A. occidentale* Extract on Male Sexual Behaviors

**Figure 1:**
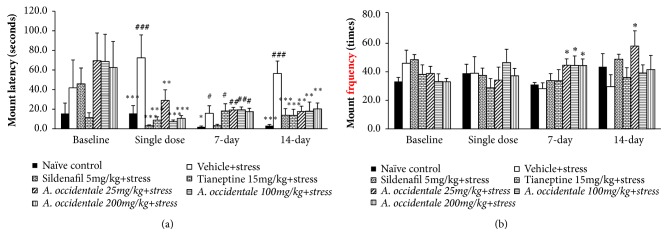
The effect of hydroalcoholic extracts of* A. occidentale* leaves extract on mount latency and mount frequency of stress exposed rats at baseline and after a single dose, 7 days, and 14 days of treatment. (a) Mounting latency. (b) Mounting frequency. Data are expressed as mean ± SEM (n=6/group). ^*∗*, *∗∗*, *∗∗∗*^*P*-value <0.05, 0.01, and 0.001, compared with vehicle plus stress. ^#, ##, ###^*P*-value <0.05, 0.01, and 0.001, compared with control group.

**Figure 2:**
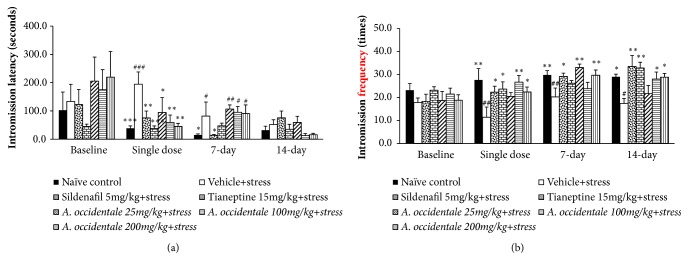
The effect of hydroalcoholic extracts of* A. occidentale* leaves extract on intromission latency and frequency of stress exposed rats at baseline and after a single dose, 7 days, and 14 days of treatment. (a) Intromission latency. (b) Intromission frequency. Data are expressed as mean ± SEM (n=6/group). ^*∗*, *∗∗*, *∗∗∗*^*P*-value <0.05, 0.01, and 0.001, compared with vehicle plus stress. ^#, ##, ###^*P*-value <0.05, 0.01, and 0.001, compared with control group.

**Figure 3:**
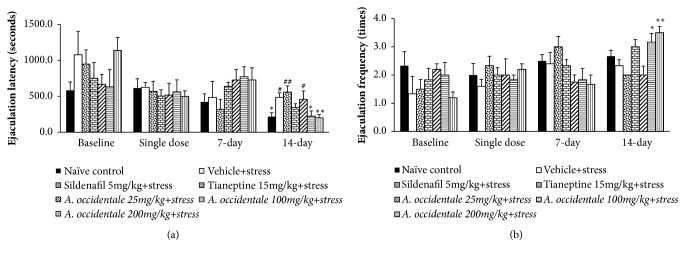
The effect of hydroalcoholic extracts of* A. occidentale* leaves extract on ejaculation latency and frequency of stress exposed rats at baseline and after a single dose, 7 days, and 14 days of treatment. (a) Ejaculation latency. (b) Ejaculation frequency. Data are expressed as mean ± SEM (n=6/group). ^*∗*^*P*-value <0.05, compared with vehicle plus stress. ^#, ##^*P*-value <0.05 and 0.01, compared with control group.

The effects of* A. occidentale* leaves extract on sexual behaviors were shown in Figures 1–3. The baseline data showed that no significant changes of all sexual parameters among various groups were observed in this study (*F*(6,32) = 1.172,* p*= .346; mount latency,* F*(6,31) = 1.099,* p*= .385; mount frequency,* F*(6,34) = 0.78,* p*= .630; intromission latency,* F*(6,35)=0.730,* p*= .629; intromission frequency,* F*(6,32)=1.047,* p*= .414; ejaculation latency,* F*(6,32)=1.034,* p*= .421; ejaculation frequency). After the single administration, stress exposed rats which received vehicle significantly increased latencies of mounting (*F*(6,34) = 6.060,* p* < .0001) and intromission (*F*(6,30) = 3.969,* p *= .005) but decreased intromission frequency (p-value < .01, compared to naïve control group). When the treatment was prolonged to 7 days, mounting and intromission latencies (*F*(6,30) = 2.954,* p* = .022), (*F*(6,30) = 2.831,* p *= .026) together with the frequency of intromission* F*(6,30) = 3.388,* p* = .011) also showed significant differences between groups. When compared to the naïve control group, the significant elevation of mounting and intromission latencies together with the reduction of intromission number was still observed (p-value<.05, .05, and .01, respectively). At 14 days of intervention period, both mounting and ejaculation latencies and intromission frequency revealed the significant differences between groups [(*F*(6,33) = 5.197,* p* = .0001), (*F*(6,32) = 4.072,* p *= .004), and (*F*(6,34) = 3.631,* p* = .007), respectively]. It was found that the stress exposed rats which received vehicle significantly increased mounting and ejaculation latencies but decreased intromission frequency (*p*-value<.001 .05, and .05, respectively, compared to naïve control group). Vehicle significantly increased mounting and ejaculation latencies but decreased intromission frequency (*p*-value<.001 .05, and .05, respectively, compared to naïve control group) in stress exposed rats.

After the single administration, the statistically significant differences between groups in mount and intromission latencies [(*F*(6,34) = 6.060,* p* = .000); (*F*(6,30) = 3.969,* p *= .005), respectively] together with intromission frequency (*F*(6,30) = 2.574,* p* = .039) were observed. LSD post Hoc Test for multiple comparisons revealed that stress exposed rats which received Sildenafil and Tianeptine significantly decreased the latencies of mount and intromission (*p*-value < .001 all,* p*-value < .01 all, respectively, compared to stress plus vehicle group) but significantly increased intromission frequency (*p*-value<.05 all, compared to stress plus vehicle group).* A. occidentale* leaves extract at doses of 25, 100, and 200 mg/kg BW also significantly decreased latencies of mounting (*p*-value<.01, .001, and .001, respectively, compared to stress plus vehicle group) and intromission (*p*-value<.05, .01, and .01, respectively, compared to stress plus vehicle group). In addition, the decreased intromission frequency induced by stress exposure was also mitigated by* A. occidentale* leaves extract both at doses of 100 and 200 mg/kg BW (p-value<.01 and .05, respectively, compared to stress plus vehicle group) after the single administration. At 7 days of treatment period, data obtained from ANOVA analysis showed that both Sildenafil and Tianeptine and all doses of* A. occidentale* leaves extract used in this study failed to modulate the elevation of mounting latency in stress exposed rats (*F*(6,30) = 2.954,* p* = .022) but significantly mitigated the elevation of intromission latency* (F*(6,30) = 2.831,* p *= .026) and intromission frequency* (F*(6,30) = 3.388,* p *= .011). Sildenafil significantly decreased intromission latency but increased intromission frequency (*p*-value < .05 all, compared to stress plus vehicle group) whereas Tianeptine failed to show the significant modification effect in stress exposed rats at 7 days of treatment. At this treatment period, it was found that stress exposed rats which received* A. occidentale* leaves extract at all doses significantly increased mounting frequency (*p*-value < .05 all, compared to stress plus vehicle group). In addition, the increased intromission frequency was observed in stress exposed rats which received* A. occidentale* leaves extract at doses of 25 and 200 mg/kg BW(*p*-value < .01 all, compared to stress plus vehicle group).

When the treatment was prolonged to 14 days of treatment, data obtained from ANOVA analysis showed the significant differences between groups in mount latency (*F*(6,33) = 5.193,* p* = .001), intromission frequency* (F*(6,34) = 3.631,* p *= .007), ejaculation latency* F*(6,32) = 4.072,* p* = .004), and ejaculation frequency* F*(6,33) = 5.047,* p* = .001). Post hoc test for multiple comparisons revealed that Sildenafil, Tianeptine and all doses of* A. occidentale* leaves extract significantly decreased mounting latency (*p*-value < .001, .001, .01, .01, and .01, respectively, compared to stress plus vehicle group). Sildenafil and Tianeptine treatments also enhanced intromission frequency (*p*-value < .01 all, compared to stress plus vehicle group). The low dose of extract produced the significant elevation in mounting frequency (*p*-value < .05, compared to stress plus vehicle group) whereas the medium and high doses of extract produced the significant increase in intromission frequency (*p*-value < .05 all, compared to stress plus vehicle group) and ejaculation frequency (*p*-value < .05 and .01, respectively, compared to stress plus vehicle group). In addition, stress exposed rats which received medium and high doses of extract significantly decreased ejaculation latency (*p*-value < .05 and .01, respectively, compared to stress plus vehicle group).

### 3.2. Effect of* A. occidentale *Leaves Extract on the Serum Testosterone and Corticosterone

**Figure 4:**
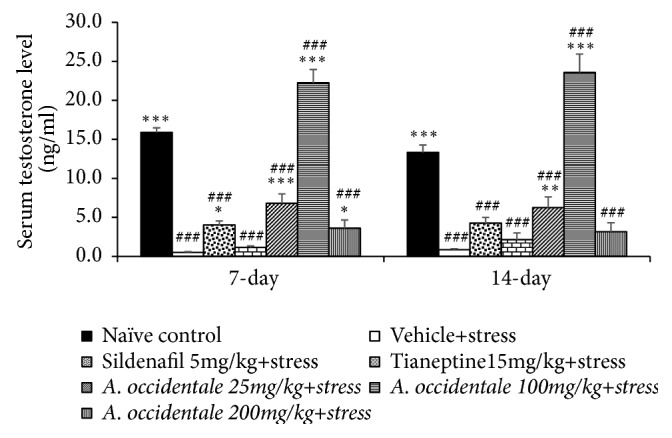
The effect of hydroalcoholic extracts of* A. occidentale *leaves extract on serum testosterone level of stress exposed rats at 7 days and 14 days of treatment. Data are expressed as mean ± SEM (n=6/group). ^*∗*, *∗∗*, *∗∗∗*^*P*-value <0.05, 0.01, and 0.001, compared with vehicle plus stress. ^###^*P*-value <0.001, compared with control group.

The effect of* A. occidentale *leaves extract on the serum testosterone level was shown in Figure 4. The significant differences in serum testosterone levels among various groups at 7 day-study period were presented (*F*(6,31) = 121.721,* p* < .0001) and 14 days (*F*(6,34) = 48.560,* p* < .0001). LSD Post Hoc test for multiple comparisons revealed that stress significantly decreased serum testosterone both at 7 and at 14 days of treatment (*p*-value <. 001 all, compared to naïve control). Sildenafil mitigated the reduction of serum testosterone in stress exposed rats at 7 days but failed to produce the significant change of this parameter at 14 days of treatment (*p*-value<.05, compared to stress plus vehicle group). However, Tianeptine failed to produce significant modification effect on this parameter in stress exposed rats throughout the study period. At 7 days of treatment, all doses of* A. occidentale *extract significantly increased serum testosterone levels (*p*-value<.001, .001, and .05, respectively, compared to stress plus vehicle group). When the treatment was prolonged further to 14 days, it was revealed that only the low and middle doses of extract significantly enhanced serum testosterone levels (*p*-value<.01 and .001, respectively, compared to stress plus vehicle group). Interestingly, the serum testosterone level of stress exposed rats which received medium dose of an extract was higher than that observed in naïve control rats both at 7 and at 14 days of treatment (*p*-value<.001 all, compared to naïve control group).

**Figure 5:**
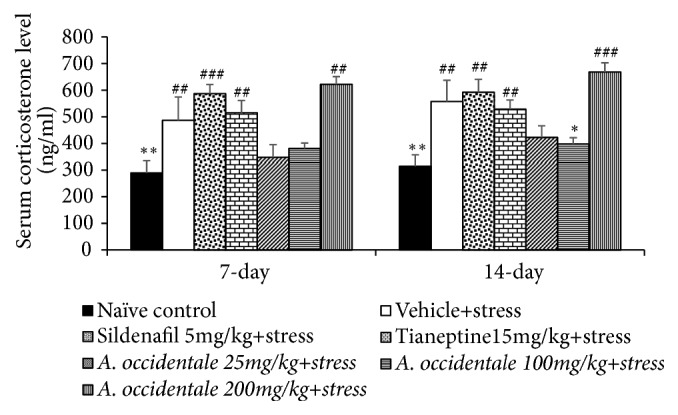
The effect of hydroalcoholic extracts of* A. occidentale *leaves extract on serum corticosterone level of stress exposed rats at 7 days and 14 days of treatment. Data are expressed as mean ± SEM (n=6/group). ^*∗*, *∗∗*^*P*-value <0.05 and 0.01, compared with vehicle plus stress. ^##, ###^*P*-value <0.01 and 0.001, compared with control group.

The effect of* A. occidentale *leaves extract on serum corticosterone, a stress hormone, was also investigated and results were shown in Figure 5. Serum corticosterone level revealed the significant differences among various groups at a 7 day-study period (*F* (6,35) =6.544,* p* < .0001) and 14 days (*F*(6,32) = 6.069,* p* < .0001) of experimental period. Stress significantly enhanced serum corticosterone level throughout the study period (*p*-value<.01 all, compared to naïve control group). Only,* A. occidentale* leaves extract at dose of 100 mg/kg BW could significantly decrease serum corticosterone level in stress exposed rats (*p*-value<.05, compared to stress plus vehicle group).

### 3.3. Effect of* A. occidentale *Leaves Extract on the Activity of PDE-5 in Penis

**Figure 6:**
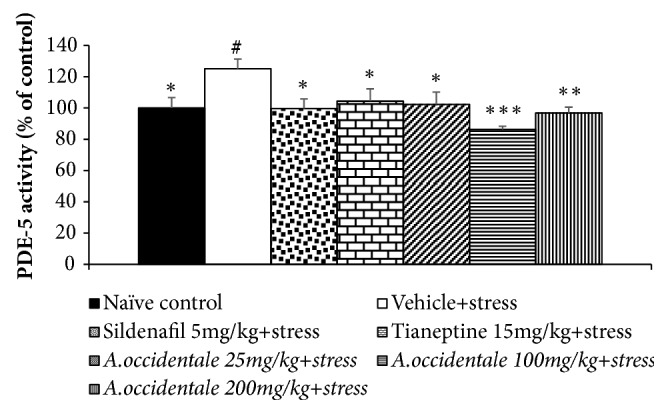
The effect of hydroalcoholic extracts of* A. occidentale* leaves extract on phosphodiesterase-5 activity in penis of stress exposed rats. Data were expressed as mean ± SEM (n=6/group). ^*∗*, *∗∗*, *∗∗∗*^*P*-value <0.05, 0.01, and 0.01, compared with vehicle plus stress. ^#^*P* -value <0,05, compared with control group.

Based on the crucial role of PDE-5 on penile erection [22], the effect of* A. occidentale *leaves extract on the activity of the mentioned enzyme in penis was also investigated and the results were shown in Figure 6. The current results showed the significant different PDE-5 activity in penis among various groups (*F*(6,34) =2.807,* p* = .025). It was found that stress increased PDE-5 activity in penis (*p*-value<.05, compared to naïve control group). However, this change was counteracted by Sildenafil, Tianeptine, and all doses of* A. occidentale *leaves extract (*p*-value<.05, .05, .05, .001, and .01, respectively, compared to stress plus vehicle group).

### 3.4. Effect of* A. occidentale *Leaves Extract on the Activity of MAO-B

**Figure 7:**
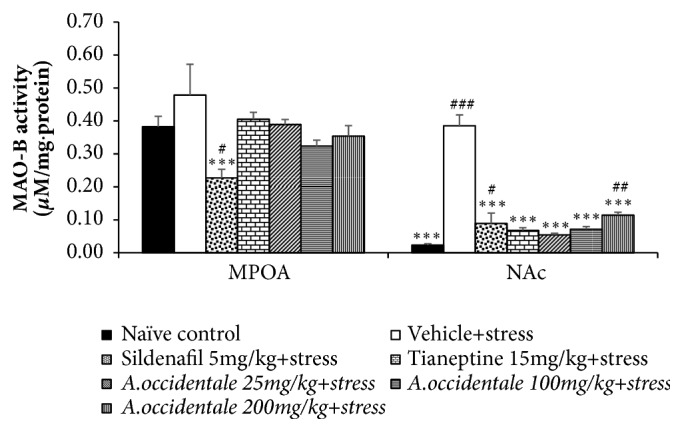
The effect of hydroalcoholic extracts of* A. occidentale* leaves extract on monoamine oxidase-B in medial preoptic area and nucleus accumbens of stress exposed rats. Data were expressed as mean ± SEM (n=6/group). ^*∗*, *∗∗∗*^*P*-value <0.05 and 0.001, compared with vehicle plus stress. ^#, ###^*P*-value <0,05 and 0.001, compared with control group.

Since dopaminergic system plays an essential role on male sexual behaviors, the effect of* A. occidentale *leaves extract on the function of dopaminergic system is considered [3]. Activity of MAO-B in medial preoptic area (MPOA) and nucleus accumbens (NAc) revealed the significant differences among various groups [(*F*(6,34) = 3.324,* p* = .011), (*F*(6,35) = 44.100,* p* < .0001).  Figure 7 demonstrated that stress exposed rats which received vehicle showed the significant elevation of MAO-B activity in nucleus accumbens (NAc) (*p*-value<.001, compared to naïve control group) but not in medial preoptic area (MPOA). It was found that Sildenafil, Tianeptine, and all doses of* A. occidentale* leaves extract could mitigate the elevation of this enzyme in NAc of stress exposed rats (p-value<.001 all, compared to stress plus vehicle group). All treatments in this study failed to modify the effect of stress on MAO-B in MPOA.

### 3.5. Effect of* A. occidentale* Leaves Extract on Histomorphology Changes of Testis

**Figure 8:**
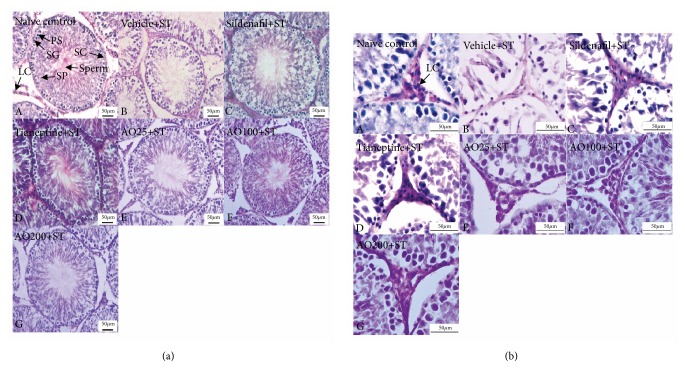
The effect of hydroalcoholic extracts of* A. occidentale* leaves extract on histomorphology of rat testis stained with haematoxylin and eosin (H&E). (a) Seminiferous tubule. (b) Interstitial cell of Leydig (n=6/group). Sertoli cells (SC), spermatogonia (SG), primary spermatocytes (PS), spermatids (SP), and sperm and Leydig cells (LC) in the following treatment groups: (A) naïve control; (B) vehicle + stress; (C) Sildenafil citrate 5mg/kg + stress; (D) Tianeptine 15mg/kg + stress; (E)–(G)* A. occidentale* at doses of 25, 100, and 200 mg/kg+stress, respectively.

Figure 8(a) showed that stress exposed rat revealed the degeneration of most seminiferous tubules including atrophied seminiferous tubules with the reduction of spermatogenic series and sperms in tubular lumen which reflected the reduction of spermatogenesis. In addition, the germinal epithelium showed disorganization as well as marked degenerative changes. It was also found that the seminiferous tubules were reduced in diameter and the interstitial spaces were increased. The changes just mentioned were mitigated by Sildenafil, Tianeptine, and all doses of* A. occidentale* leaves extract. The alteration of interstitial cell of Leydig was also assessed and results were shown in Figure 8(b). The current results showed that stress exposed rats which received vehicle showed the reduction of interstitial cell of Leydig density. This change was also attenuated by Sildenafil, Tianeptine, and all doses of* A. occidentale* leaves extract.

### 3.6. Effect of* A. occidentale *Leaves Extract on Dopaminergic Neurons

**Figure 9:**
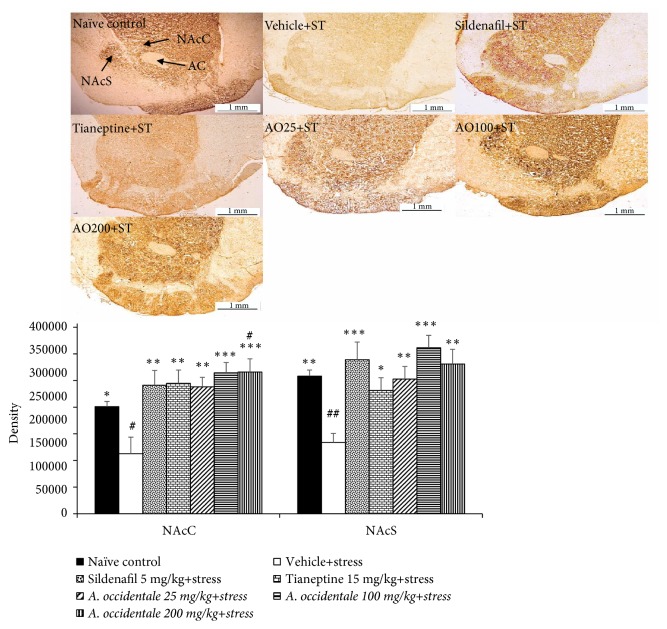
The effect of hydroalcoholic extracts of* A. occidentale* leaves extract on tyrosine hydroxylase immunoreactive neurons in core and shell of nucleus accumbens of stress exposed rats. Data were expressed as mean ± SEM (n=6/group). ^*∗*, *∗∗*, *∗∗∗*^*P*-value <0.05, 0.01, and 0.001, compared with vehicle plus stress. ^#, ##^*P*-value <0,05, and 0.01 compared with control group.

Based on the increased dopaminergic function in NAc in* A. occidentale *leaves extract treated group in this study, we also determined the effect of extract on dopaminergic neuron both in core and shell subregions of nucleus accumbens (NAcC, NAcS) and results were shown in Figure 9. The significant differences in dopaminergic neuron density in NAcC (*F*(6,33) = 5.159,* p* = .001) and NAcS (*F*(6,33) = 5.486, p < .0001) among various groups were observed. It was found that stress exposed rats significantly decreased density of tyrosine hydroxylase- (TH-) positive neuronal cell in NAcC and NAcS (*p*-value<.05 and .01, respectively, compared to naïve control group). Sildenafil and Tianeptine could attenuate the reduction of TH-positive neuronal cell in NAcC (*p*-value<.01 all, compared to stress plus vehicle group) and NAcS (p-value<.001 and .05, respectively, compared to stress plus vehicle group). All doses of extract used in this study significantly enhanced TH-positive neuronal cell density especially in NAcC (p-value<.01, .001, and .001, respectively, compared to stress plus vehicle group). Interestingly, the density of TH-positive neuronal cell in stress exposed rats which received high dose of* A. occidentale *leaves extract was higher than that in naïve intact control group (*p*-value<.05, compared to naïve control group). The increased TH-positive neuronal cell density in NAcS was also observed in stress exposed rats which received* A. occidentale *leaves extract at all doses used in this study.

**Figure 10:**
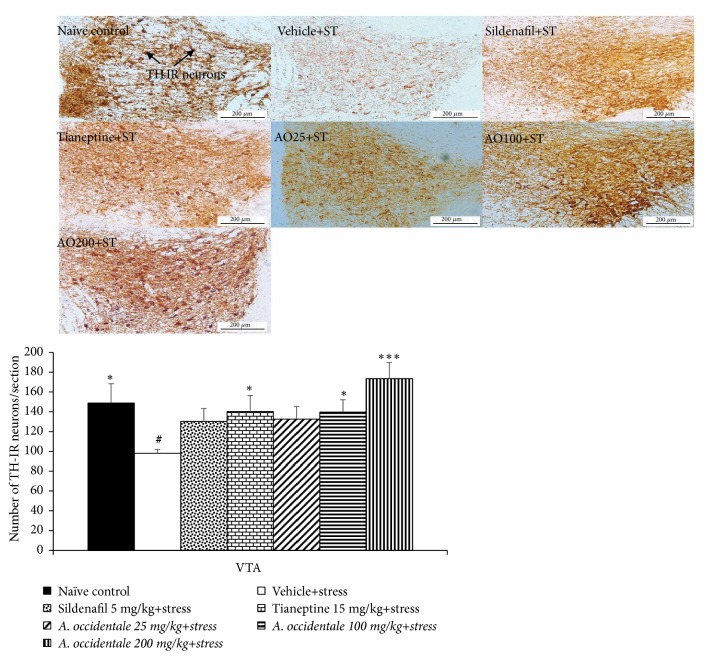
The effect of hydroalcoholic extracts of* A. occidentale* leaves extract on tyrosine hydroxylase immunoreactive neurons in vental tegmental area of stress exposed rats. Data were expressed as mean ± SEM (n=6/group). ^*∗*, *∗∗∗*^*P*-value <0.05 and 0.001, compared with vehicle plus stress. ^#^*P*-value <0,05, compared with control group.

The effect of extract on TH-positive neuronal cell density in ventral tegmental area (VTA) was also assessed due to its critical role on the regulation of male sexual behaviors [3] and results were shown in Figure 10. The current data showed the significant differences in dopaminergic neuron density in VTA among various groups (*F*(6,32) = 2.470, p = .045). Stress exposed rats which received vehicle also decreased TH-positive neuronal cell density in VTA (*p*-value<.05, compared to naïve control group). However, this change was mitigated by Tianeptine and both medium and high doses of* A. occidentale *leaves extract (*p*-value<.05, .05, and .001, respectively, compared to stress plus vehicle group).

### 3.7. Effect of* A. occidentale* Leaves Extract on eNOS Expression in Penis

**Figure 11:**
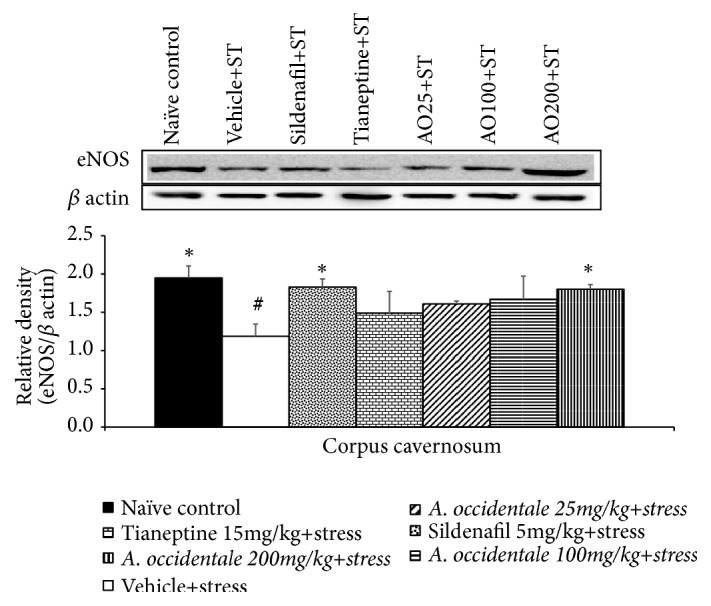
The effect of hydroalcoholic extracts of* A. occidentale* leaves extract on endothelial nitric oxide synthase in penis of stress exposed rats. Data were expressed as mean ± SEM (n=4-5/group). ^*∗*^*P*-value <0.05, compared with vehicle plus stress. ^#^*P*-value <0,05, compared with control group.

Figure 11 demonstrated the effect of* A. occidentale* leaves extract on eNOS expression in penis. The significant differences in eNOS expression in penis among various groups was observed (*F*(6,29) = 2.755,* p* = .030). It was found that stress significantly decreased eNOS expression in penis (p-value<.05, compared to naïve control group). This change was mitigated by Sildenafil and high dose of* A. occidentale* leaves extract (p-value<.05 all, compared to stress plus vehicle group).

## 4. Discussion

The current data is the first study which demonstrated the modulation effect of* A. occidentale* leaves extract on neuroendocrine control circuit of male sexual behaviors. It has clearly demonstrated that* A. occidentale* leaves extract enhanced male sexual behaviors, suppressed MAO-B activity in both mPOA and NAc, and suppressed corticosterone level in stress exposed rats. In addition, the elevation of serum testosterone level and the enhanced density of dopaminergic neurons in NAc and VTA together with the increased eNOS were also observed in stress exposed rats which received* A. occidentale* leaves extract.

Sexual arousal also contributes an important role on male sexual behavior especially in precopulatory behavior. The mesocorticolimbic DA tract which ascends from the ventral tegmental area (VTA) to the nucleus accumbens (NAc) and prefrontal cortex is reported to play a critical role on reinforcement and appetitive behaviors. However, various subregions of NAc exert different roles. The nucleus accumbens core (NAcC) is involved in the cognitive processing of cognitive processing of motor function related to reward and reinforcement whereas the nucleus accumbens shell (NAcS) is involved sexual performance, reward-related processing, and the inhibition of sexual behavior after ejaculation [23].

Copulatory behavior is also under the influence of testosterone. Testosterone especially metabolites of testosterone are essential for the dopaminergic function especially in MPOA. The low level of DA appears to exert disinhibition of genital reflex via the increase in parasympathetic nervous system leading to penile erection whereas the high dose of DA increase seminal emission and ejaculation [24].

Under normal circumstance, dopaminergic system plays the crucial role on the regulation of male sexual behavior [3] and emotion regulation such as anxiety [25]. However, anxiety is a complex phenomenon and depends not only on dopamine but also on GABA and serotonin [26]. Our results showed that the extract can enhance the activity of dopaminergic system in the mesolimbic area especially in nucleus accumbens (NAc), medial preoptic area (mPOA), and ventral tegmentum area (VTA). In addition, the suppression of MAO-B in NAc was also observed. Therefore, the extract can modulate both the male sexual functions and the regulation of mood including anxiety. Since the negative emotions decreased sexual arousal or libido [27], the indirect modulation effect on male sexual behaviors of the extract had been considered. In addition, imaging study had revealed that the actions of gamma amino butyric acid (GABA) and serotonin could decrease the mesolimbic dopaminergic system [26]. However, anxiolytic drugs such as benzodiazepines which stimulate GABAergic system significantly decreased libido and increased erectile and orgasm dysfunction [28]. In addition, no scientific evidence concerning the suppression of PDE-5, and the enhancements of eNOS, spermatogenesis, and density of interstitial cell of Leydig presented until now. Based on aforementioned information, we did suggest that the extract might exert the effect to enhance the male sexual performance partly via its direct effect on the increase in mesodopaminergic activity in NAc and VTA which in turn enhanced mounting, intromission and ejaculation [3, 29]. Interestingly, the density of dopaminergic neuron in NAcC in stress exposed rats which received high dose of the extract is higher than naïve control group. These data suggested that the extract might be able to increase neurogenesis. However, this requires further investigation. In addition to the enhancement of mesolimbic dopaminergic system, the extract also could also the increase interstitial cell of Leydig in testis and the elevation of testosterone. Aforementioned changes also contributed an important role on the enhancement of male sexual function. The suppression of PDE-5 and the increase in eNOS activity induced by the extract treatment could also contribute the role especially in penile erection. The negative correlation between stress hormone and male sexual function [30] was also reported. Therefore, the extract could also decrease stress hormone and induced the improvement of male sexual behavior. However, only the changing of mesolimbic dopaminergic activity showed the close relationship between the extract doses and the improvement of male sexual parameters. Therefore, the increase in mesolimbic dopaminergic system might be served as the main pathway in modulating male sexual function in stress exposed rats which received extract treatment. The suppression of PDE-5 contributed an important role especially on penile erection [31]. In addition, the elevation of testosterone also played a role in the low and medium doses of* A. occidentale* leaves extract.

This study failed to show the dose dependent manner because all observed parameters were associated with the multifactor and the masking effect of inactive ingredients could also contribute the role. Based on the pivotal roles of polyphenol on the suppression of MAO-B [32] and its role on penile erection via eNOS [33] together with the improved testicular functions [34] induced by polyphenol rich substance, we did suggest that the sexual enhancing effect of* A. occidentale* leaves extract might be associated with polyphenolic compounds content in the extract. However, this still required further investigation.

Taken all data together, sexual enhancing effect of* A. occidentale* extract occurred mainly via the improved dopaminergic function via the increase in dopaminergic neuron in VTA and NAc and the suppression of MAO-B together with the improved testicular function. In addition, PDE-5 suppression effect in penis also contributed the role especially in the increased intromission behavior. The current data also suggested that the enhanced eNOS activity might play a role at high dose whereas the decreased corticosterone might play a role at the medium dose of extract.

## 5. Conclusion

**Figure 12:**
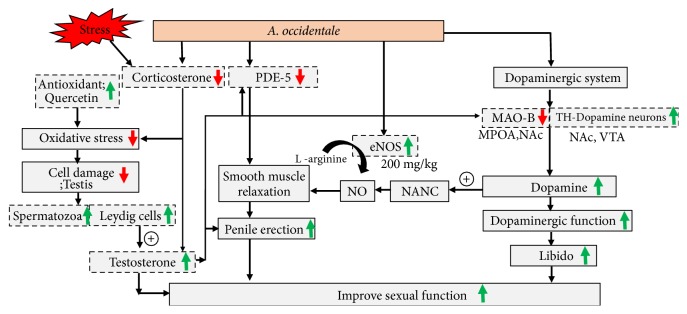
Schematic diagram showed the possible underlying mechanism of* A. occidentale* on sexual performance in stress exposed rats.

*A. occidentale* leaves extract is the potential functional ingredient for enhancing male sexual function. In addition, it also has the potential to protect against male sexual dysfunction and infertility induced by stress. The possible underlying mechanism might occur mainly via the improved dopaminergic function via the increase in dopaminergic neuron in VTA and NAc and the suppression of MAO-B together with the improved testicular function. In addition, PDE-5 suppression effect in penis also contributed the role especially in the increased intromission behavior as shown in Figure 12. Therefore,* A. occidentale* leaves extract can be served as the functional ingredient for developing food supplement targeting at improving male sexual dysfunction and infertility induced by stress.

## Data Availability

The data used to support the findings of this study are available from the corresponding author upon request.
